# Research on the open space ratios of residential plots in major Chinese cities meeting public centralized green space standards

**DOI:** 10.1038/s41598-025-10282-w

**Published:** 2025-10-06

**Authors:** Bingjing Zhao, Xu Liu, Fang Yan, Chen Wang, Minghua Huang

**Affiliations:** 1 School of Architecture, Xi’an University of Architecture and Technology, Xi’an, 710055 China; 2 Zhengzhou University of Aeronautics, Zhengzhou, 450015 China

**Keywords:** Open space ratio, Public centralized green space standards, Land use and development control, Major cities, Residential plots, Environmental sustainability, Environmental social sciences, Environmental impact, Sustainability

## Abstract

The indicator of open space ratio has garnered significant international attention due to its ability to effectively balance equity and efficiency in urban development. In China, many studies have investigated the introduction of the open space ratio to address the disconnect between public benefit factors and development efficiency factors during the land development control process. However, these studies have not yet succeeded in linking the open space ratio with the core element of public interest—public centralized green spaces—resulting in an inability to further localize the open space ratio within the Chinese context. To address the gaps in existing research, our study focuses on residential plots in 15 major cities across different climate zones in China, exploring the open space ratios that meet the country’s standards for public centralized green spaces. We find that, after achieving standards, the open space ratios in residential plots across various cities have increased to varying degrees. The differences in open space ratios among specific cities are significantly influenced by factors such as geographical latitude, the intensity of per capita land use, regional economic development levels, and local construction practices. The application of the open space ratio with Chinese characteristics must be based on the premise of meeting the public interest baseline. It should be tailored to local conditions, stratified and classified, and scientifically determined, thereby promoting an effective balance between equity and efficiency in the urban land development process and advancing the sustainable development of the living environment.

## Introduction

As China’s economy transitions from a phase of rapid growth to one focused on quality enhancement, establishing a baseline for spatial development and meeting the public’s demand for a high-quality living environment are essential requirements for achieving equitable, efficient, and sustainable urban development. Regulatory detailed planning serves as the legal basis for land use control in China, allocating land development rights through a system of assigned indicators. It plays a crucial role in maintaining market development order, safeguarding public interests, and enhancing the quality of the spatial environment. Essentially, it functions as a set of “rules” with universal binding force on various construction activities^1^. However, as urban land development demands become increasingly diverse and complex under a market-oriented approach, issues such as weak constraints, insufficient consensus, and frequent adjustments in regulatory detailed planning have become more pronounced. This is particularly evident in the drafting stage, where the core indicator system centered on land development control—primarily focusing on floor area ratio—emphasizes economic efficiency and lacks a direct connection to public interest factors related to environmental quality, such as mandatory sunlight distances and public centralized green spaces outlined in relevant standards^2^. Additionally, the green space ratio is primarily influenced by building density and road area, without a direct correlation to per capita green space^3^. The lack of constraint from public interest baselines, such as sunlight access and green space, has led to a heavy reliance on subjective experience during the drafting of regulatory plans. This results in insufficient flexibility when addressing complex demands, while frequent adjustments contribute to a rigidity that undermines the stability of "control." Consequently, the universal binding nature of these “rules” is weakened, leading to an exacerbation of imbalances between equity and efficiency in the land development process^1,2,4,5^. This, in turn, compromises the quality of the spatial environment and hinders the achievement of national goals for land space development and protection that aim for "higher quality, greater efficiency, enhanced equity, and improved sustainability."^6^.

The Open Space Ratio (OSR) is a core regulatory indicator for residential land development control in New York City, USA. It maintains a positive proportional relationship between the area of open space and total building area within zoning lots, thereby safeguarding residents’ equal rights to a healthy living environment^2,4,5,7–10^. The OSR not only ensures a reasonable relationship between urban public interests and market development benefits, but also allows for greater flexibility in market development and architectural design by guaranteeing a basic open space ratio regardless of development intensity. This approach enables a more “universal” and “precise” control that scientifically balances equity and efficiency, offering enhanced stability. If this indicator were introduced into China’s regulatory detailed planning and adapted to the national context, it could serve as the foundation for a land development control indicator system that includes a range of rigid and flexible indicators. Coupled with appropriate expression methods for the results, this could explore a "rule-based" theory and methodology for urbanl land development control that reconciles equity and efficiency, fundamentally promoting the effective role of regulatory plans as "rules."^2,5,8^

International research on the Open Space Ratio (OSR) primarily focuses on two areas: theoretical understanding and practical exploration. (1) Theoretical Understanding: On one hand, existing research emphasizes the “rule” attribute of the OSR from a theoretical perspective, positioning it as a key link between land development benefits and public interests. Controlling the OSR can effectively influence urban land economics, housing density, and environmental quality, promoting the optimal allocation of land resources^11–14^. On the other hand, theoretical discussions surrounding the OSR primarily focus on its role in urban spatial analysis. Various studies highlight its connection to urban design and spatial planning, with the OSR being viewed as a key parameter for managing the economic and social dimensions of urban development. In particular, the relationship between OSR and urban spatial density has been explored, making significant theoretical contributions^15–18^. For instance, the Spacemate model by Berghauser Pont and P. Haupt positions the OSR as a key indicator for measuring urban spatial density, linking it to other metrics such as the Floor Space Index (FSI), Ground Space Index (GSI), and average building height (L). This theoretical framework contributes to a deeper understanding of the relationships among urban spatial forms, land use, and environmental quality^16–18^. (2) Practical Exploration: Practically, the OSR has been applied across various urban contexts, demonstrating its versatility in urban planning and development. Applications include urban development on sloped terrain^19^, high-rise building site design^20,21^, and the revitalization of historic districts^22–24^. Additionally, OSR has been used in urban infrastructure optimization^25^, development around Transit-Oriented Development (TOD) stations^26,27^, and the creation of public spaces^28–30^. Furthermore, international scholars from countries such as Spain, the Netherlands, France, Russia, and the United Kingdom have applied OSR in measuring urban spatial density, showing its global relevance^31–35^. Besides, a key aspect of OSR research is its use in urban spatial analysis, particularly with GIS-based methods. Many studies have utilized Geographic Information Systems (GIS) to measure and analyze OSRs as part of broader urban spatial density assessments^2,5,10,14,16–18,22,36^. GIS provides an efficient means of quantifying spatial characteristics and understanding urban morphology. For example, GIS has been used in analyzing residential land plots, calculating OSR values, and mapping open spaces in urban environments^2,36^. However, it should be noted that while the GIS is a commonly used tool in urban spatial analysis, its role in existing research has largely been limited to calculating and spatially representing the OSR values. To further analyze the OSR in conjunction with other public interest factors, such as sunlight and green spaces, it is necessary to integrate additional software tools.

**Fig. 1:**
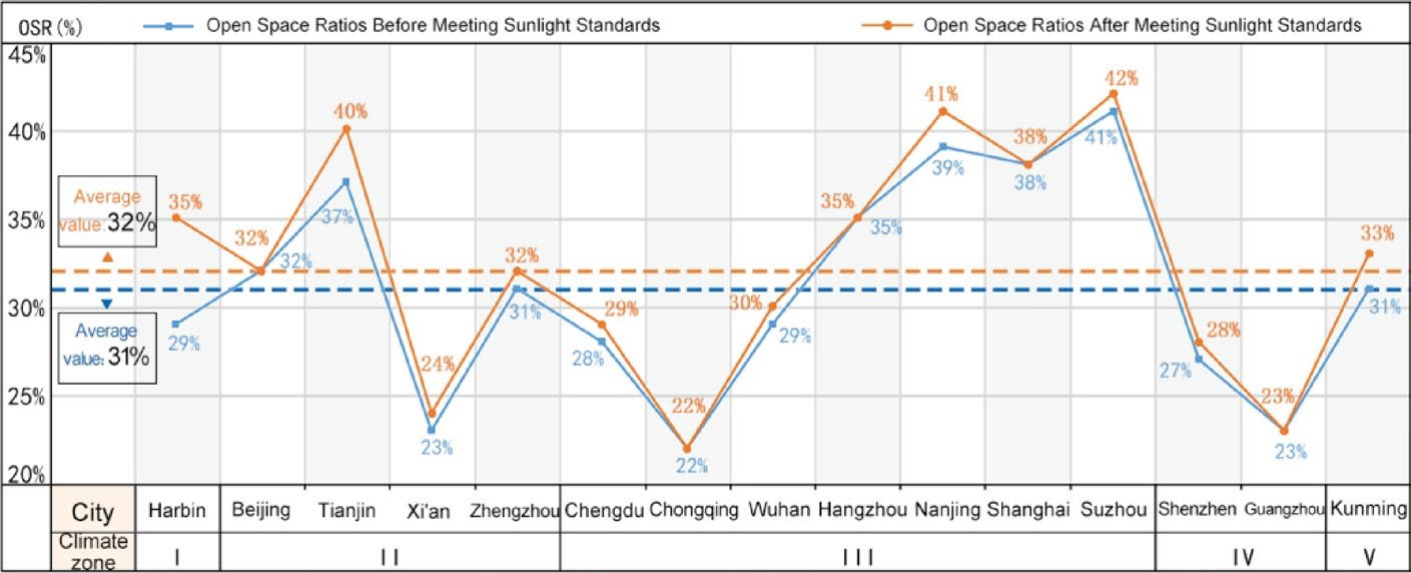
Changes in the overall average open space ratio of residential plots in cities after meeting sunlight standards. Data Source: References [2, 5].

**Fig. 2:**
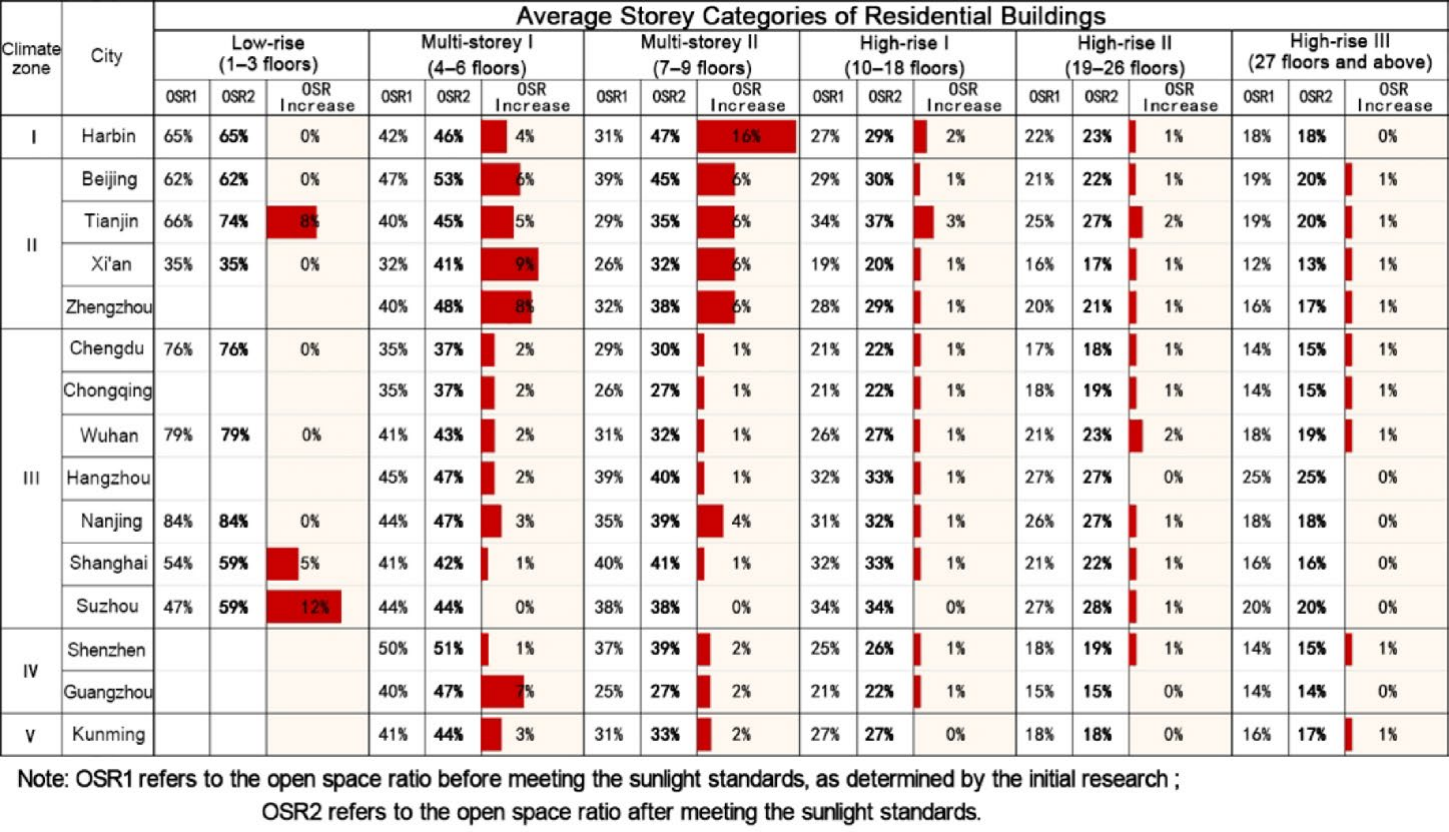
Changes in the average open space ratio of residential plots with different average storey categories in cities after meeting sunlight standards. Data Source: References [2, 5].

In China, scholars are increasingly delving into research on the Open Space Ratio (OSR), leading to a clearer understanding of its concept and implications^1,2,5,7,8,36^. Some studies have proposed utilizing the OSR in urban land development control. Our team conducted a survey on the current state of OSR in residential plots ranging from 2 to 4 hectares in typical Chinese cities. We found that the OSR in residential plots is inversely proportional to development intensity, with an overall average of 31%^5^. Based on these findings, our research team further explored OSR values in relation to China’s residential building sunlight standards. We discovered that, after meeting these standards, the OSR in urban residential plots showed varying degrees of improvement, significantly influenced by factors such as the level of economic development in the region, sunlight conditions, and residential layout configurations (Figs. 1 and 2)^2^.

Although existing studies have investigated the basic characteristics of open space ratios (OSRs) in residential plots of typical Chinese cities and explored changes in OSRs values after meeting sunlight standards, sunlight is merely a foundational factor for ensuring the quality of the residential environment. For residential plots, public centralized green spaces play a more significant and direct role in influencing the quality of the public environment. These green spaces provide essential venues for residents’ public activities, effectively promote physical and mental health, and serve as a core element in achieving high-quality living environments. Existing research has not systematically studied the relationship between OSRs and public centralized green spaces within the context of Chinese cities. In prior datasets, only residential plots meeting sunlight standards were included, without further consideration of the need for public centralized green spaces. As a result, some plots that do not meet green space requirements were included, making it difficult to fully align OSR findings with the construction of public interests.

To address these gaps, this study builds upon existing work and employs a comprehensive approach. First, public data on typical residential plots were collected through field surveys, interviews with urban planning authorities, and integration of data from national travel mapping platforms. These data were processed and analyzed using GIS to calculate OSR values. Next, residential plots were analyzed for sunlight accessibility, and only those meeting the sunlight requirements were selected. Finally, public centralized green space factors were incorporated to investigate the OSRs of residential plots in major Chinese cities, with a focus on changes in OSR values after meeting public centralized green space standards. Specifically, this study addresses the following research questions:

- How do OSRs vary across residential plots with different average storey categories and different building layout forms?
- How do OSRs vary across representative cities within different climate zones?
- What factors contribute to these variations?
- How can OSRs guide urban planning to ensure a balance between public interest and development efficiency?

In summary, this study aims to comprehensively characterize the OSR of residential plots in Chinese cities that meet public interest requirements. By doing so, it seeks to establish a foundation for developing application strategies and methodologies for OSRs tailored to China’s national context. The study also supports the rational determination of baseline OSR values with Chinese characteristics, contributing to rectifying the imbalance between fairness and efficiency in existing development control practices.

## Materials and methods

### Research objects

**Fig. 3:**
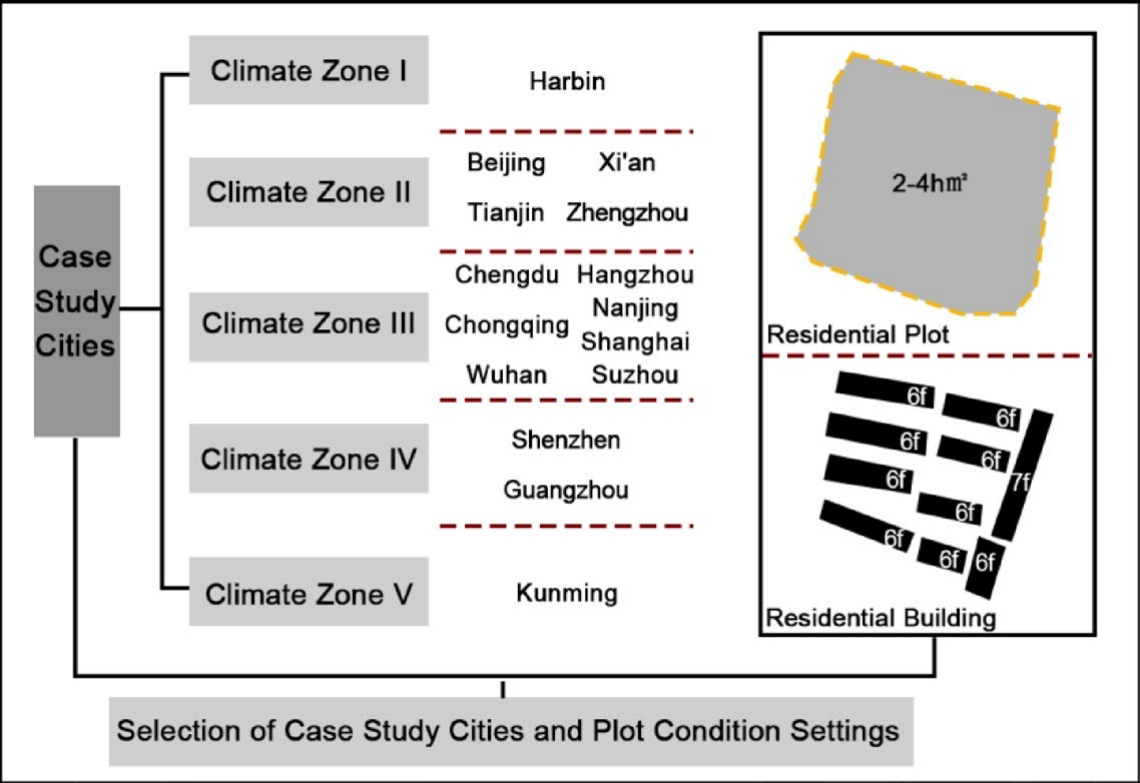
Research subjects and conditions setting. *Source*: Reference [5].

**Fig. 4:**
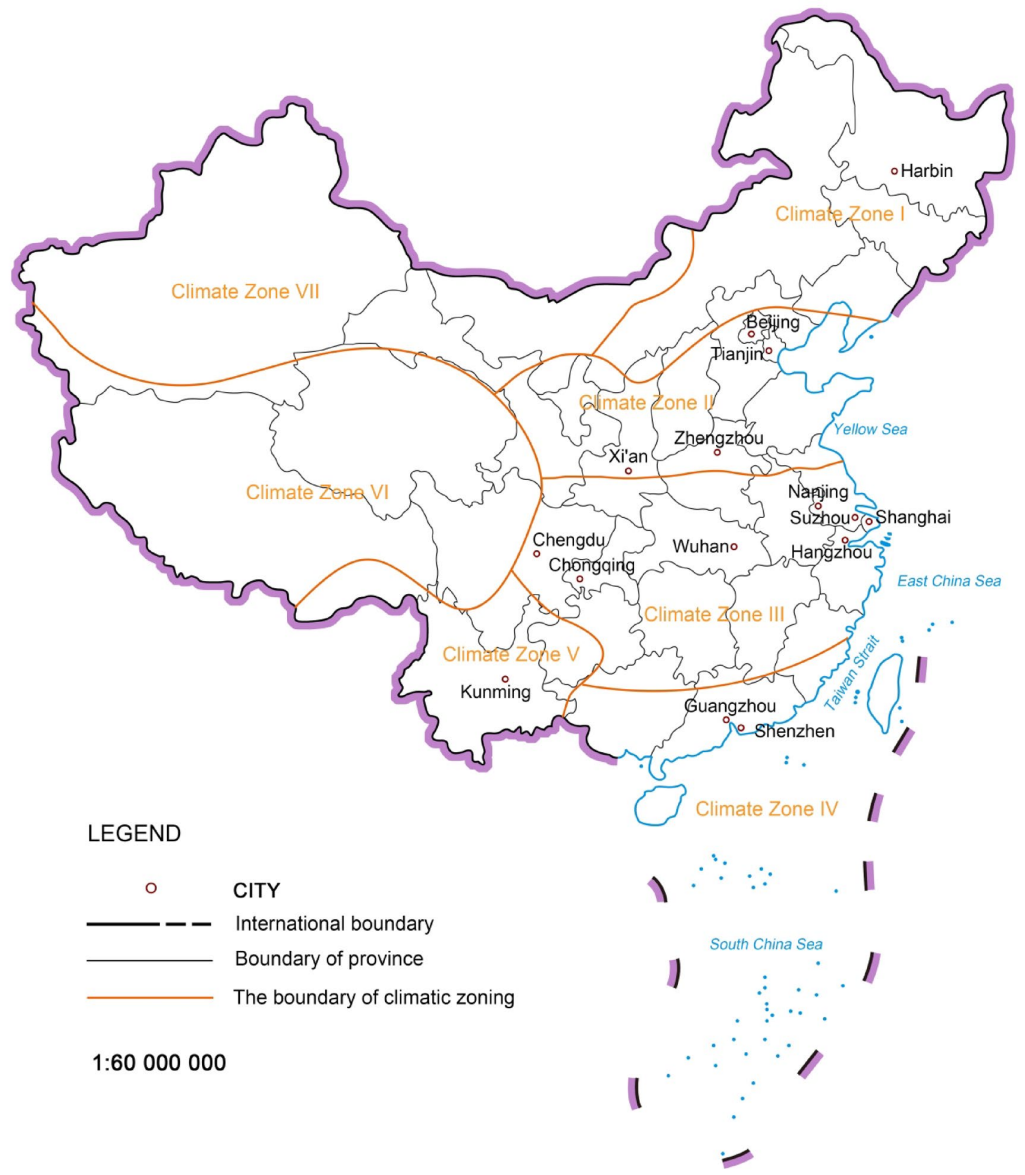
Distribution of case study cities. Map source: http://bzdt.ch.mnr.gov.cn.

The research focuses on residential plots ranging from 2 to 4 hectares in major cities of China. First, residential land constitutes the largest proportion of urban construction land and directly impacts the interests of every resident. In practical planning and management processes, conflicts of interest among various stakeholders in residential areas are particularly concentrated, leading to frequent imbalances between equity and efficiency^37,38^. Therefore, this research examines residential plots, selecting an area of 2 to 4 hectares based on the "Planning and Design Standards for Urban Residential Areas" (GB 50,180–2018)^39^ issued by China. Second, considering that the footprint and building area of ground-level commercial buildings are relatively small and have minimal overall impact on the Open Space Ratio (OSR) of residential plots, and that their scope is difficult to define, this study excludes the influence of ground-level commercial building volume. Open space is defined as the area outside the residential buildings, calculated as the difference between the residential plot area and the footprint of the residential buildings. Finally, regarding the source cities of the plots, the research acknowledges that the planning and construction of residential plots are influenced by climate. Based on China’s architectural climate zoning, representative cities were selected from climate zones I to V, which have higher population densities. Harbin was selected from Climate Zone I; Beijing, Xi’an, Tianjin, and Zhengzhou were selected from Climate Zone II; for Climate Zone III, which spans a wide area, the study selected Chengdu, Chongqing, and Wuhan from central and western China, as well as Hangzhou, Nanjing, Shanghai and Suzhou from the southeastern Yangtze River Delta region (Figs. 3 and 4). These cities are representative of the region and were selected for analysis in this study due to their significant urban development and relevance to the research topic.

### Methods

#### Data sources

To address the inadequacy of existing research in considering public interest factors, this study uses data that meets sunlight standards from previous research as the foundational data source^2,5,8^, allowing for further data filtering in conjunction with Public centralized green space standards. In this process, data for residential plots ranging from 2 to 4 hectares in case study cities was collected from multiple sources, including field surveys, interviews with urban planning authorities, and integration of data from the National Travel Mapping Platform. This multi-source approach ensures comprehensive coverage and enhances the reliability of the data. Specifically, the National Travel Mapping Platform selected was Baidu Maps (https://map.baidu.com/), an official navigation application widely used in China. Baidu Maps offers global geographic information services such as smart positioning, AOI (Area of Interest) retrieval, route planning, navigation, and real-time traffic conditions. The use of Baidu Maps facilitates the identification of residential parcels from an overall urban perspective. Many studies have also utilized Baidu Maps for the extraction of geographic data in China^40–44^. This study focuses on residential land, retrieving information on 2 to 4-hectare residential parcels and their building AOI from the API interface of Baidu Maps. Ultimately, the study collected data on a total of 8,572 plots, with building information that includes residential land use and building floor count.

**Fig. 5:**
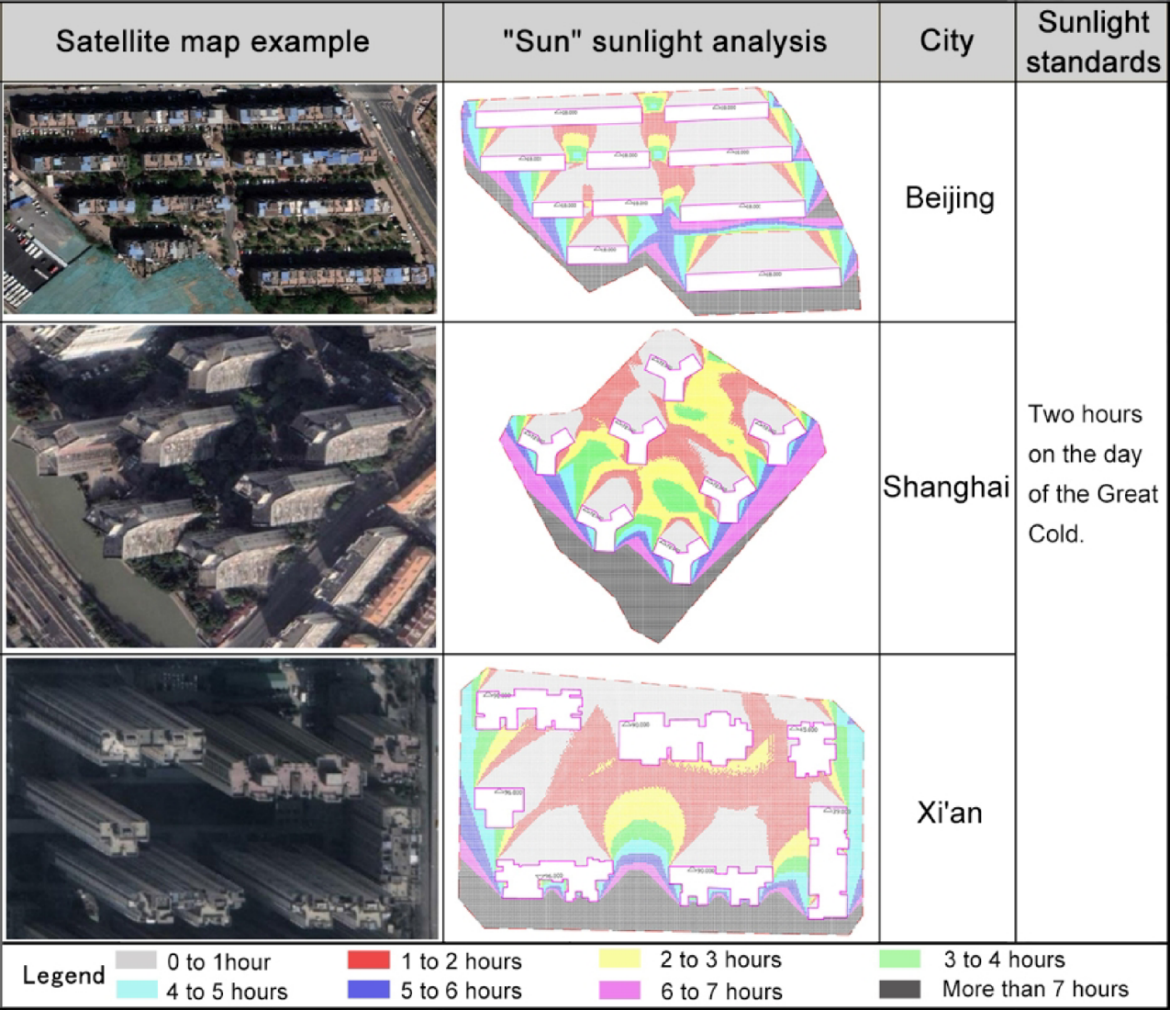
Example of sunlight analysis for residential parcels. *Source*: Reference [2], plotting results combined from Baidu Maps (https://map.baidu.com/) and SUN sunlight analysis software.

Meanwhile, due to the inconsistency in construction timelines across different residential plots in cities, and considering the underdeveloped state of sunlight analysis technology in early China, some residential plots do not meet the required sunlight spacing standards. To address this, the study integrates the sunlight standards specified in China’s "Standards for Planning and Design of Urban Residential Areas" (GB50180-2018) (Table 1)^39^ and employs the "SUN Sunlight Analysis" software, which runs within AutoCAD, to conduct sunlight analysis on residential plots. This software was selected for its detailed and localized simulation of sunlight conditions, specifically tailored to urban residential plots in China (Fig. 5). Compared to more generic sunlight analysis tools, "SUN Sunlight Analysis" provides higher accuracy in representing local sunlight conditions, which is crucial for meeting the sunlight spacing requirements in residential development. Based on the analysis results from this software, the study further filtered residential plots that meet the sunlight standards from the initial survey data, yielding a total data source of 6,036 entries (Table 2)^2,5,8^.

**Table 1:** Residential building sunlight standards.

Climate zoning	I, II, III, VII		IV		V, VI
Permanent urban population (in ten thousand)	≥ 50	< 50	≥ 50	< 50	Unlimited
Sunlight standard day	The day of the Great Cold			The day of the Winter Solstice	
Sunlight duration (h)	≥ 2	≥ 3		≥ 1	
Effective sunlight duration zone (local true solar time)	8:00 to 16:00			9:00 to 15:00	
Calculation starting point	Ground-level window sill

**Table 2:** Number of data sources in each city. Data source: References [2, 5, 8] ^2,5,8^

Climate zone	Typical city	Number of residential parcels	Data source:number of parcels meeting sunlight standards
I	Harbin	302	94
II	Beijing	636	379
	Tianjin	601	223
	Xi’an	450	258
	Zhengzhou	373	200
III	Chengdu	1017	800
	Chongqing	649	532
	Wuhan	466	278
	Hangzhou	481	416
	Nanjing	480	330
	Shanghai	1669	1437
	Suzhou	255	213
IV	Shenzhen	527	398
	Guangzhou	356	269
V	Kunming	310	209
Total		8572	6036

### Data screening for compliance with public centralized green space standards

1. Screening criteria
  To facilitate the conduct of the study, this research follows the relevant provisions of the "Planning and Design Standards for Urban Residential Areas" (GB50180-2018) (Fig. 6)^39^ regarding centralized green spaces in residential plots. Based on the analysis results from the "SUN Sunlight Analysis" software, the study assesses whether the open space of residential plots meets the requirements for public centralized green space allocation. The green space compliance status is calculated for a total of 6036 data sources.

**Fig. 6:**
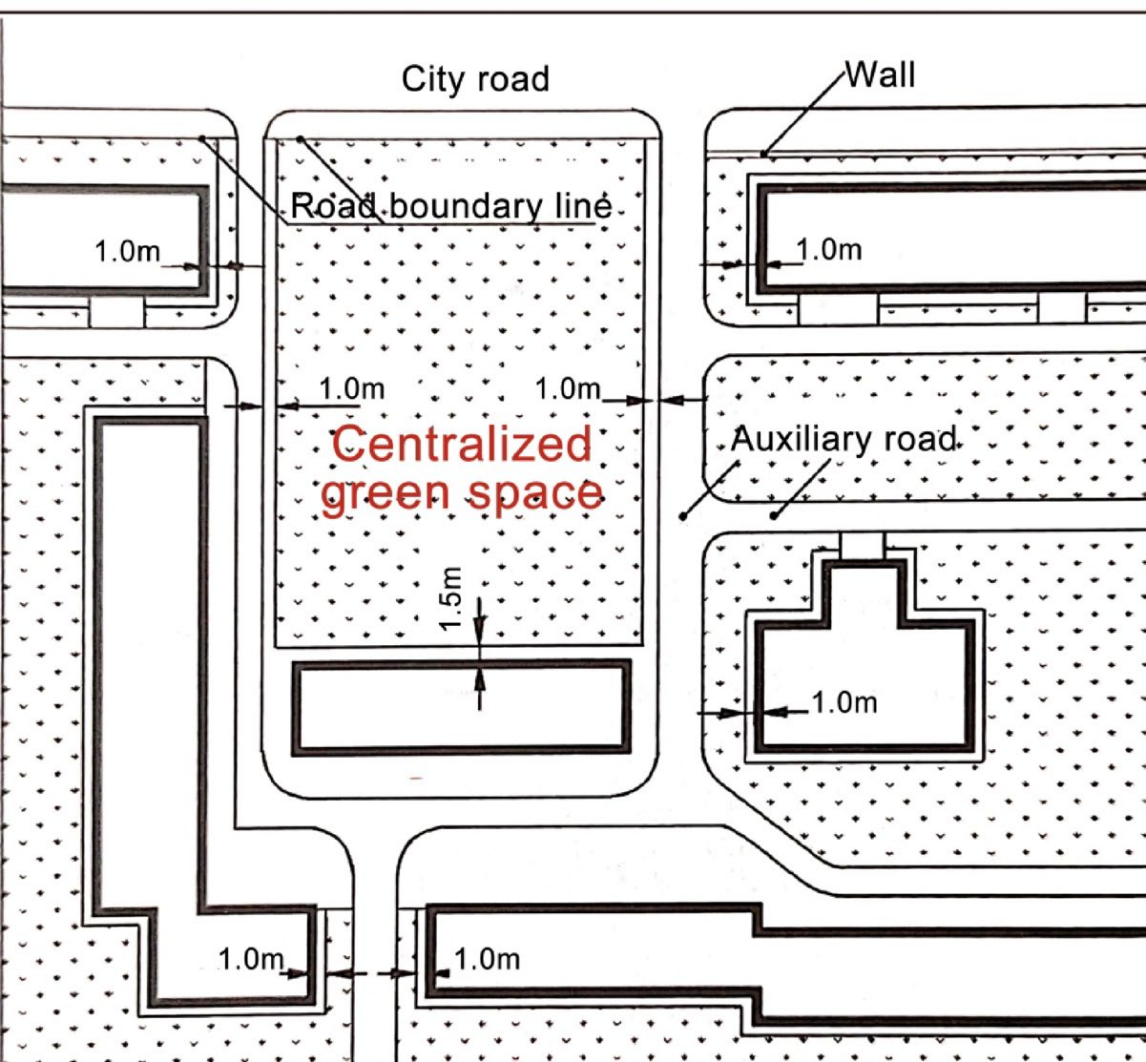
Illustrative rules for calculating public centralized green space within residential Plots. *Source*: Planning and design standards for urban residential areas (GB50180-2018).
  i. Per capita public centralized green space area^39^
    The construction of new districts should not be less than 0.5 m^2^ per person, while the redevelopment of old districts should not be less than 0.35 m^2^ per person.
  ii. Requirements for public centralized green space area under sunlight^39^
    At least one-third of the green space area must be outside the shadow line defined by the building’s daylighting standards.
2. Data filtering and organization for meeting public centralized green space standards
  i. Due to the significant influence of residential building layouts on the arrangement of public centralized spaces within residential plots, this study categorizes the original plots into three types based on building types, in addition to the classification by average number of stories: slab-type residential plots, slab-point combined residential plots, and point-type residential plots^45–47^ (Table 3).

**Table 3:**
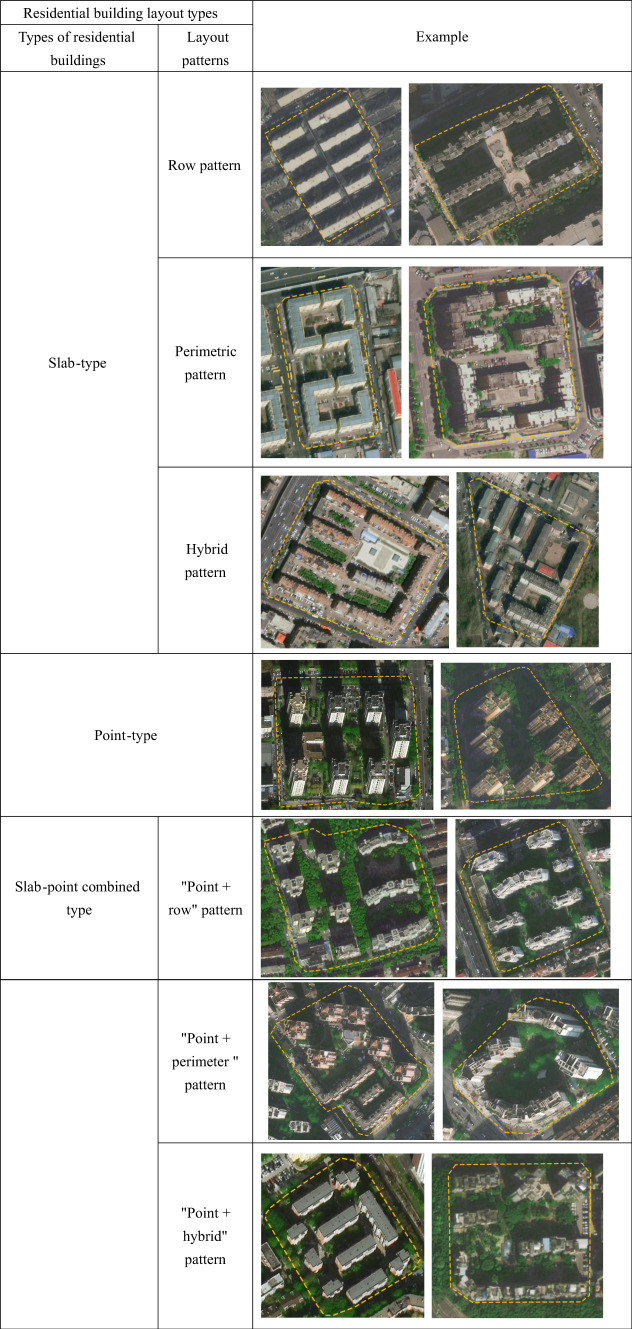
Classification of residential plot types
  First, the slab-type residential plots are composed of residential buildings that have a wide front facing south, a short depth, north–south ventilation, and an overall flat appearance. Based on different layout patterns, slab-type residential plots can be further divided into three types: row pattern, perimetric pattern, and hybrid pattern that combines elements of both row and perimetric designs. The row pattern refers to a grouping of buildings arranged in rows with a specific orientation and reasonable spacing. The perimetric pattern involves buildings arranged along roads, creating a closed or semi-closed courtyard space (Table 3).
  Secondly, point-type residential plots consist of tower-style buildings. These tower residential buildings generally have comparable depth, width, and dimensions, and are typically arranged around a central stairway hub, accommodating several households (Table 3).
  Thirdly, slab-point combined residential plots include both slab-type and point-type buildings. The layout patterns of these plots can be categorized into three types: "point + row" pattern、"point + perimeter " pattern and "point + hybrid" pattern (Table 3).
3. According to the regulations regarding public centralized green spaces in the "Standards for Planning and Design of Urban Residential Areas," and in conjunction with the collected population data and map measurements, this study evaluates whether residential plots that meet sunlight standards also comply with the construction standards for public centralized green spaces. Ultimately, residential plots that fulfill the requirements for sunlight, green space, and other public interests are identified and selected. The data filtering and organization approach is as follows (Fig. 7).

**Fig. 7:**
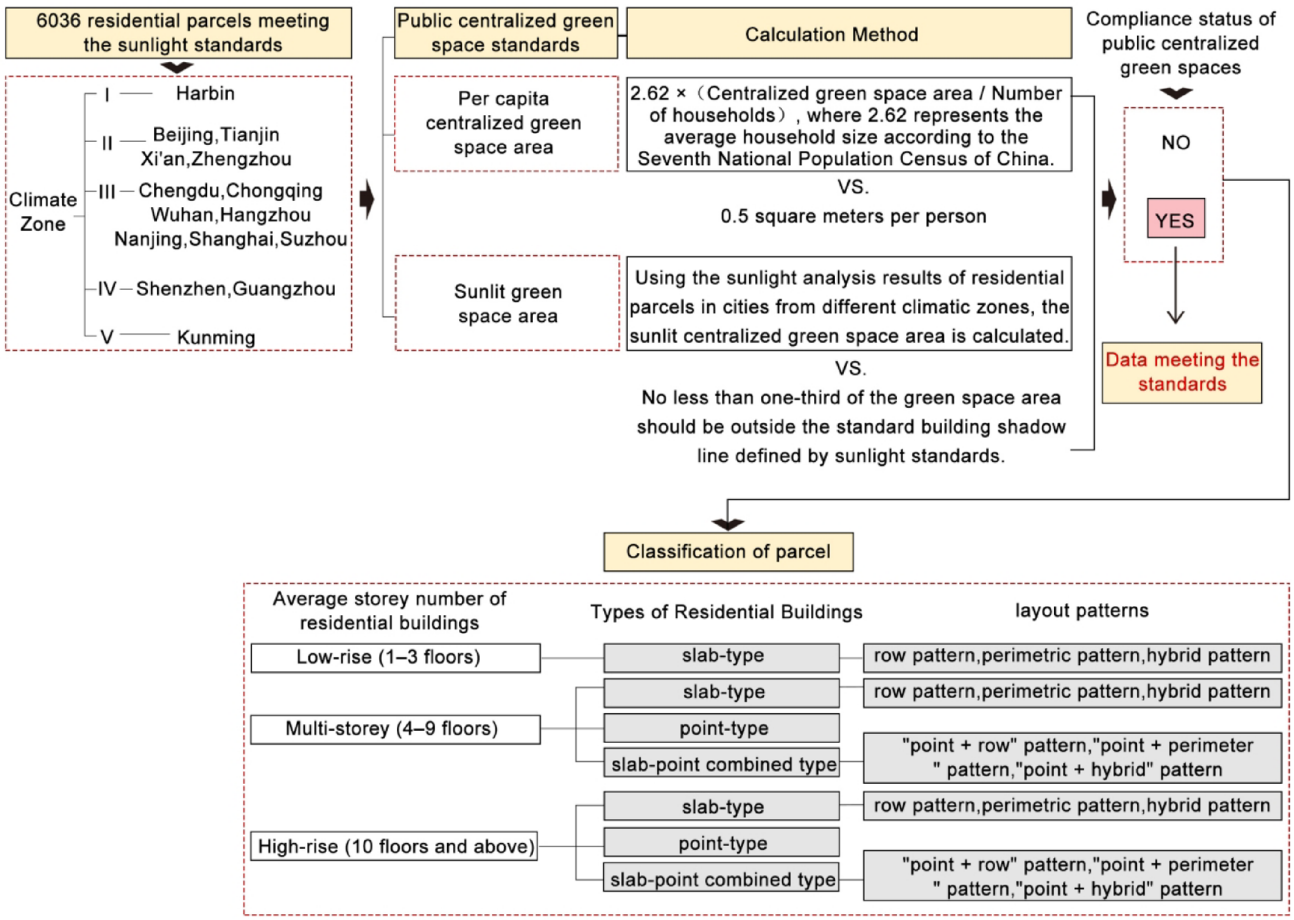
Data filtering and organization approach.

### Open space ratio calculation

Open space refers to the area on a parcel of land that is not occupied by buildings. The open space ratio (OSR) indicates the proportion of open space area to the total building area on that parcel^5,7,9^.

1 $$ \begin{array}{*{20}c} {OSR = 100\% \times \frac{{A_{OS} }}{{A_{b} }}} \\ \end{array} $$

In this equation, *OSR* represents the open space ratio, which indicates the percentage of open space relative to the total building area. *A*_*os*_ is the area of open space, and *A*_*b*_ refers to the total building area.

The equation can be further expanded as follows:

2 $$ \begin{array}{*{20}c} {OSR = 100\% \times \frac{{A_{S} - A_{bf} }}{{A_{b} }}} \\ \end{array} $$

In this version, A_s_​ stands for the site area, which is the total land area of the parcel. A_bf_ represents the building footprint area, which is the ground area covered by the building. A_b_​ still refers to the total building area.

The OSR is inversely related to another core indicator of residential development control, which is the Floor Area Ratio (FAR).

3 $$ \begin{array}{*{20}c}    {FAR = \frac{{A_{b} }}{{A_{S} }}\frac{{A_{b} }}{{A_{{OS}} }}}  \\   \end{array}  $$

In this equation, FAR represents the Floor Area Ratio. A_b_ is the total building area, A_s_ is the site area, and A_os_ is the area of open space. The expression indicates that the floor area ratio is inversely related to the area of open space.

This can also be expressed as:

4 $$ \begin{array}{*{20}c}    {FAR = \frac{{A_{b} }}{{A_{S} }}\frac{1}{{A_{{OS}} }}}  \\   \end{array}  $$

Here, the reciprocal of the area of open space A_os_ shows the inverse relationship between the floor area ratio and the open space area.

The interrelationship between the Open Space Ratio (OSR), Floor Area Ratio (FAR), and average storey number of residential buildings can be expressed as follows:

5 $$ \begin{array}{*{20}c} {OSR = 100\% \times \frac{{A_{S} }}{{A_{b} }} - \frac{{A_{bf} }}{{A_{b} }}} \\ \end{array} $$

In this formula, A_s_ represents the site area, which is the total area of the land parcel, A_b_ is the total building area, and A_bf_ refers to the building footprint area, which indicates the area occupied by the building on the ground.

This equation can also be expressed as:

6 $$ \begin{array}{*{20}c} {OSR = 100\% \times \frac{1}{{{\text{FAR}}}} - \frac{{A_{bf} }}{{{\text{n}} \cdot A_{b} }}} \\ \end{array} $$

In this version, FAR represents the Floor Area Ratio, and n denotes the average number of storeys in residential buildings. The term A_bf_ is still the building footprint area, and A_b_​ remains the total building area.

Based on the above mathematical relationship, it can be observed that when the average storey number of residential buildings on a site is fixed, a higher FAR leads to a lower OSR, indicating an inverse relationship between the two. When the FAR on a site is fixed, the OSR increases as the average storey number of residential buildings increases. Conversely, when the OSR on a site is fixed, both the average storey number and the FAR may increase simultaneously. Therefore, there is a certain interdependent “pulling” effect between the OSR, FAR, and the average storey number of residential buildings, which allows the alignment of economic benefits with public interest^5,8^.

The study employs geographic information systems (GIS) to calculate OSR of residential plots in selected cities. GIS was chosen due to its effectiveness in spatial data processing and its ability to accurately compute OSR values across large datasets, which is crucial for large-scale analysis of urban residential areas. The study, based on the formula for calculating the OSR, first analyzes the average OSR of residential plots before and after meeting the public centralized green space standards, categorized by the average storey number of residential buildings and the type of residential building. This analysis is then visualized using bar charts for data presentation. Next, the study focuses on specific cities to examine the changes in OSR before and after meeting the public centralized green space standards, summarizing the influencing factors behind the changes in these indicators.

## Result

### The compliance status of urban residential plots with standards

#### Overall compliance status

The statistical results indicate that the overall compliance rate of public centralized green space standards in residential plots across major cities in China is 73%.

**Fig. 8:**
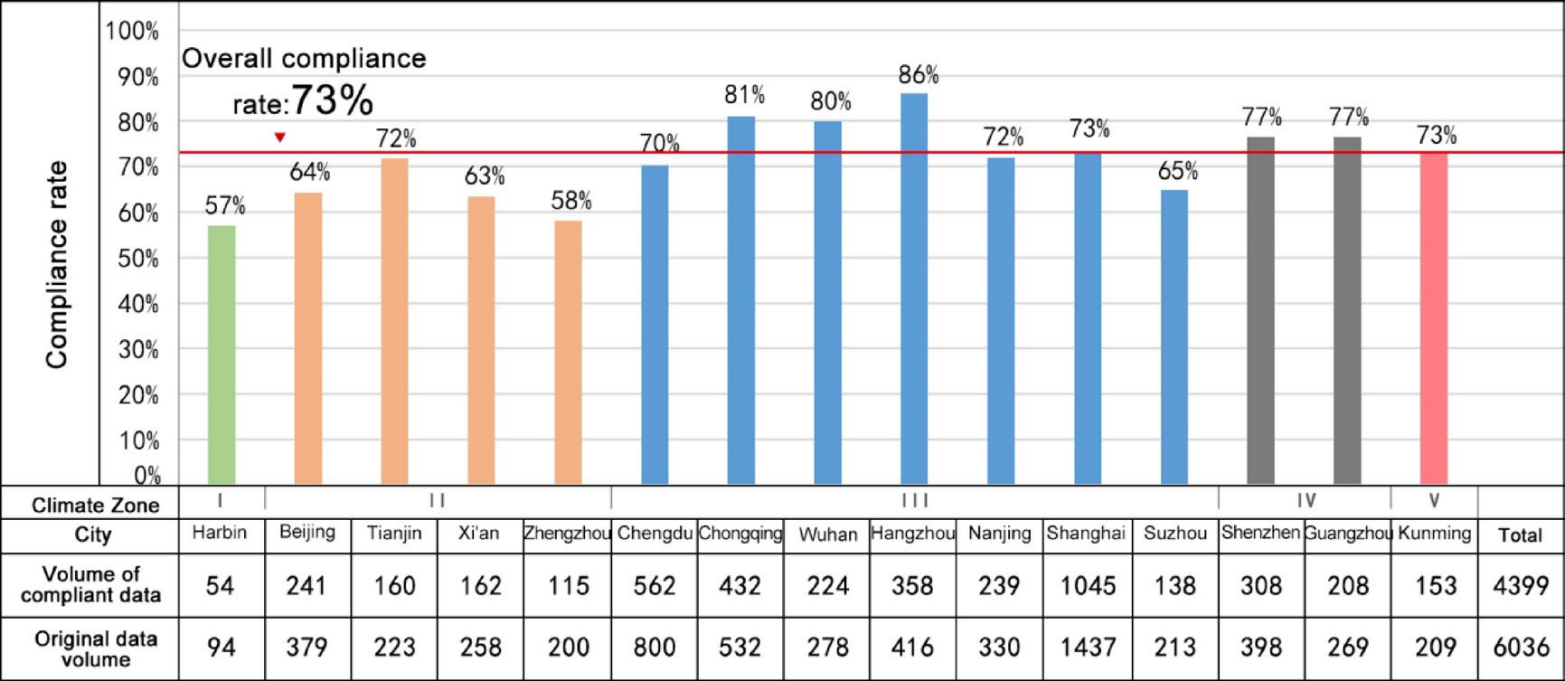
The overall statistics on the compliance status of public centralized green space standards in residential plots across major cities in China.

The compliance rate of public centralized green space in residential plots located in Climate Zones I–II is below the overall average. Among these cities, Harbin has the lowest compliance rate, at only 57%. In contrast, the compliance rates in cities located in Climate Zones III–V are generally higher, with most exceeding the overall average. Hangzhou has the highest compliance rate at 86%, while Suzhou, which belongs to the same climate zone as Hangzhou, has a relatively lower compliance rate of only 65% (Fig. 8).

#### Compliance status of different categories of residential plots

**Fig. 9:**
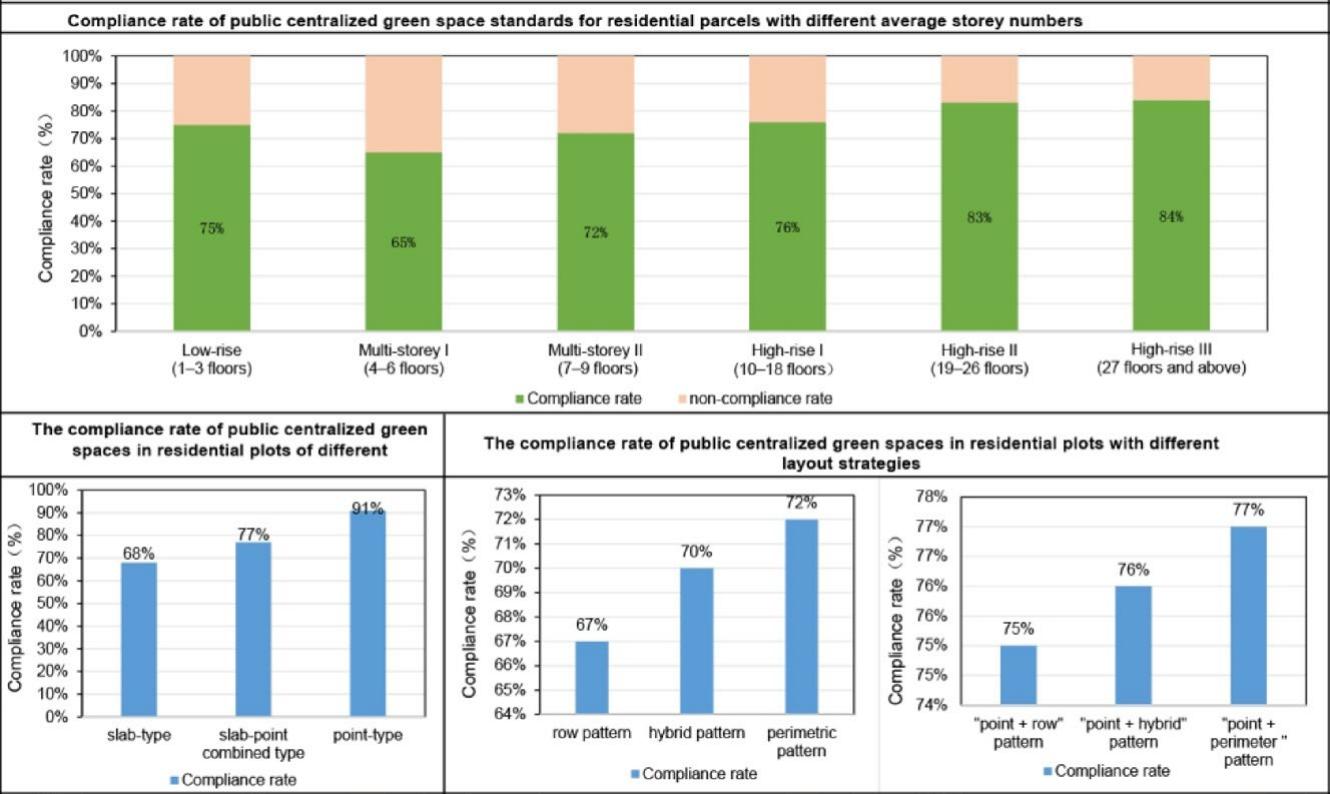
Compliance rate of public centralized green space in different types of residential plots.

The compliance rates of public centralized green spaces in residential plots categorized by different average storey numbers of residential buildings and various architectural forms exhibit distinct characteristics (Fig. 9).

1. Compliance status of residential plots with different average storey categories
  Low-rise residential plots, due to their lower development intensity and smaller building shadow coverage over centralized green spaces, have relatively sufficient green space. Combined with the smaller resident population, the per capita satisfaction with green space is high. From multi-storey to high-rise categories, the compliance rate of public centralized green spaces in residential plots shows an increasing trend as the average storey category rises. This is because multi-storey residential plots typically only meet sunlight spacing requirements, with compact layouts and higher building densities, resulting in limited space for public centralized green areas. Additionally, multi-storey plots often experience significant building shadow coverage, leading to insufficient sunlight exposure on the green areas and, consequently, lower compliance rates. However, as the average storey category increases, the sunlight spacing of residential buildings expands, and both the area of centralized green space and the proportion of green space outside the standard building shadow range increase. Therefore, compared to multi-storey residential plots, high-rise plots, despite their higher development intensity, still provide sufficient green space to meet the per capita demand for centralized green spaces, resulting in a higher compliance rate for public centralized green spaces.
2. Compliance status of residential plots with different building layout forms
  In terms of residential building types, the highest compliance rate for green spaces is observed in point-type residential plots, followed by mixed point-and-slab type, with slab-type residential plots having the lowest green space compliance rate. Point-type buildings have smaller shadow coverage in all orientations, larger building spacing, and flexible layouts that facilitate the arrangement of centralized green spaces, leading to a higher compliance rate for green spaces. However, when the development intensity of point-type residential plots is high, there may be insufficient per capita green space, resulting in non-compliance with green space standards.
  Slab-type buildings, with their wider façades and smaller lateral spacing between buildings, tend to cast significant shadows on centralized green spaces. Many developers, aiming to maximize land economic benefits, only meet the sunlight spacing requirements without considering the need for centralized green space arrangement, resulting in less than one-third of the green space falling outside the standard building shadow range. Consequently, slab-type residential plots have lower compliance rates for public centralized green spaces. This misunderstanding of “building spacing” as "centralized green space" is particularly evident in row pattern layouts.

In contrast, perimetric pattern layouts, which often use more east–west oriented residential buildings to create centralized open spaces, cause less shadowing on green areas. Therefore, compared to row pattern layouts of the same storey category, perimetric pattern layouts are more likely to meet public centralized green space standards, resulting in higher compliance rates.

Hybrid pattern layouts, which combine characteristics of both row and perimeter styles, have a compliance rate for public centralized green spaces that falls between the two. Plots with combined point-and-slab type layouts, which incorporate characteristics of both slab-type and point-type buildings, also exhibit compliance rates between the two. In terms of layout strategies, the "point + perimeter " pattern combination has the highest compliance rate, followed by the "point + hybrid" pattern, with the "point + row" pattern "combination having the lowest compliance rate.

### Overall characteristics of the open space ratio in residential plots meeting public centralized green space standards

#### Open space ratio of residential plots with different average storey categories

After meeting the public centralized green space standards, the average OSR of residential plots across different average storey categories increased to varying degrees. Among them, low-rise residential plots saw the highest increase in the average OSR (5%), followed by multi-storey plots (2–3%), while high-rise plots experienced the lowest increase (1%).

**Fig. 10:**
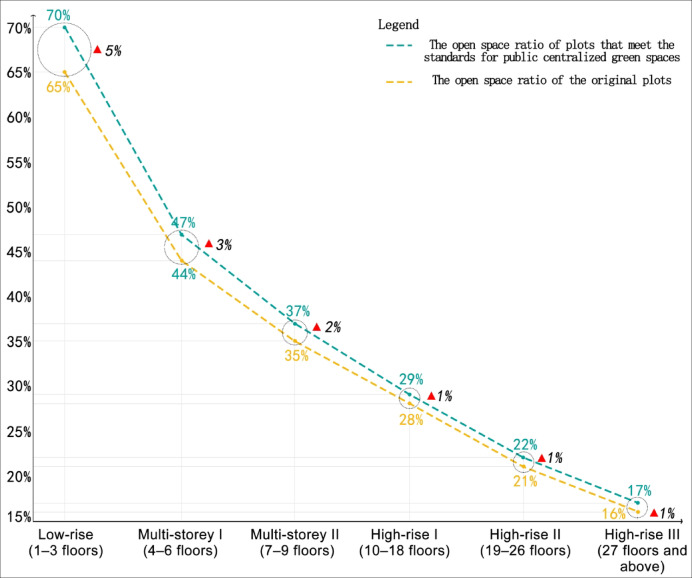
The increase in the open space ratio of residential plots categorized by different average storey numbers of residential buildings after meeting the standards for public centralized green spaces.

The underlying reason is that low-rise residential plots tend to exhibit a polarized distribution of building density, with either very high or very low density. The non-compliance with centralized green space standards is often found in plots that only meet the minimum sunlight spacing requirements, without reserving space for centralized green areas, resulting in poor environmental quality. Consequently, low-rise residential plots show the largest increase in the average OSR after meeting green space standards. Compared to high-rise plots, multi-storey residential plots require more open space to meet centralized green space construction requirements, leading to a greater reduction in building density and a higher increase in the OSR (Fig. 10).

#### Open Space ratio of residential plots with different building layout forms

1. Open space ratio of residential plots with different residential building types
  Firstly, after meeting the public centralized green space standards, among multi-storey and high-rise residential plots, slab-type residential plots experienced the highest increase in OSR, followed by combined slab-and-point type, with point-type residential plots seeing the lowest increase. The reason is that slab-type residential plots need to create significantly more open space than point-type plots to meet the standards, leading to a greater reduction in building density and, consequently, a higher increase in the OSR. The increase for combined slab-and-point plots lies between the two. Secondly, from multi-storey to high-rise categories, the average OSR increase for slab-type and combined slab-and-point residential plots gradually decreases. Compared to multi-storey point-type residential plots, high-rise point-type plots, due to higher development intensity, require larger building spacing to meet the public centralized green space standards, resulting in a higher increase in the OSR (Fig. 11).

**Fig. 11:**
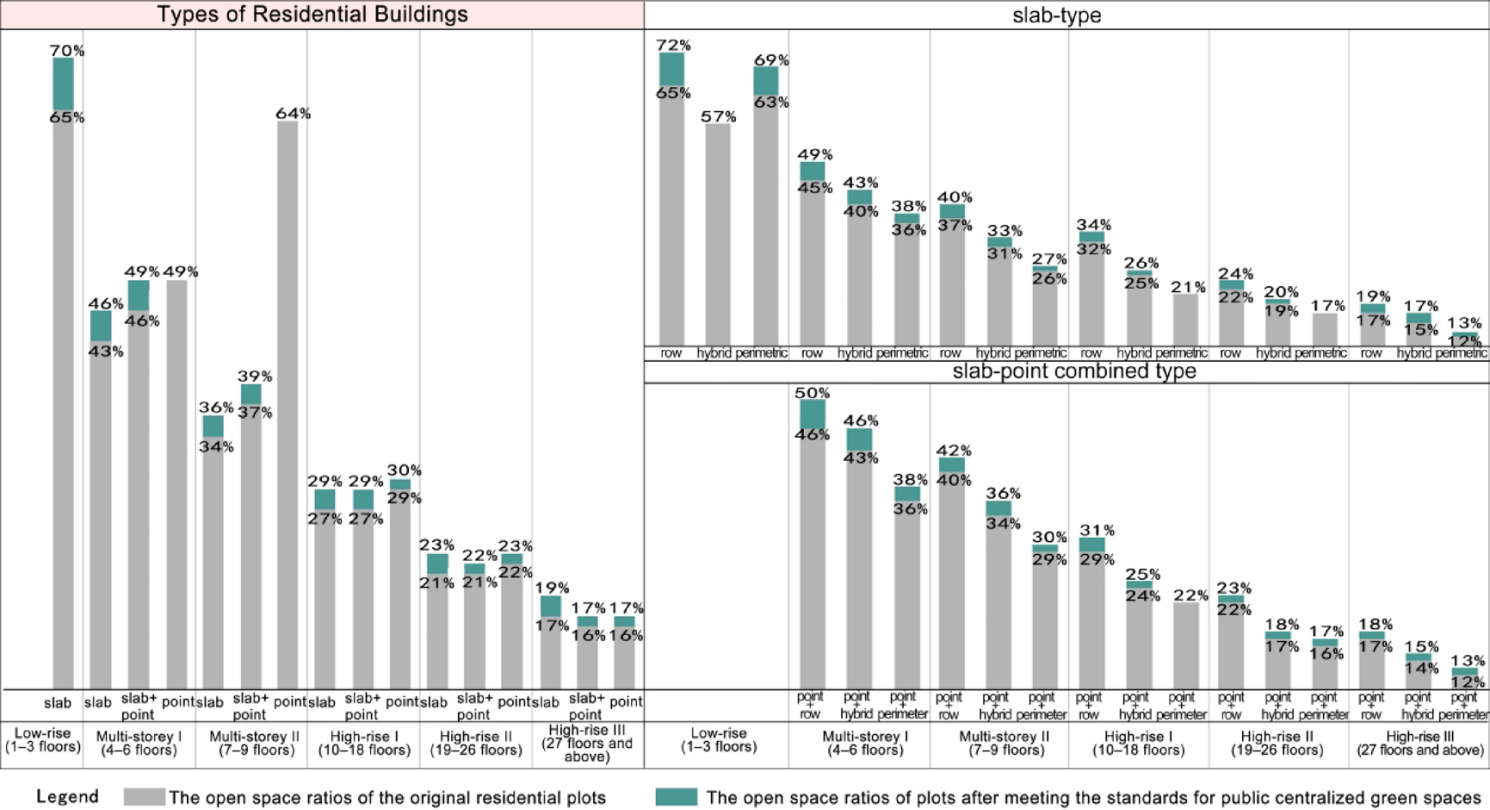
The variation in the average open space ratio of residential plots with different building layouts after meeting the standards for public centralized green spaces.
2. Open space ratio of residential plots with different layout methods
  Among slab-type residential plots, those with row pattern layouts have the highest OSR, followed by hybrid patterns, with perimetric pattern layouts having the lowest. The findings from our field surveys reveal that, compared to row pattern layouts, compared to the typically north–south oriented row-pattern layouts, the perimetric residential layout further incorporates east–west oriented housing units in the vertical direction. This modification enhances land use efficiency, which results in increased development intensity and consequently a lower OSR. Hybrid pattern plots, which combine the characteristics of both row and perimetric patterns, have an OSR that falls between the two.
  In terms of changes in the OSR for slab-type plots after meeting green space standards, row pattern layouts see the greatest increase in the OSR, followed by hybrid patterns, with perimetric pattern layouts experiencing the smallest increase. This is because, in the same storey category, row pattern plots need to create significantly more open space than perimetric pattern plots to meet the public centralized green space standards, leading to a greater reduction in building density and a higher increase in the OSR.
  For combined point-and-slab type residential plots of the same average storey category, the OSR exhibits similar characteristics to slab-type plots. Specifically, "point + row" pattern residential plots have the highest OSR, followed by "point + hybrid" pattern, with "point + perimeter" pattern plots having the lowest. Regarding the increase in the OSR after meeting green space standards, "point + row" pattern plots see the largest increase, followed by "point + hybrid" pattern, with "point + perimeter" pattern plots experiencing the smallest increase (Fig. 11).

### Open space ratio of residential plots in major cities meeting green space standards

#### Open space ratio of residential plots with different average storey categories in cities

**Fig. 12:**
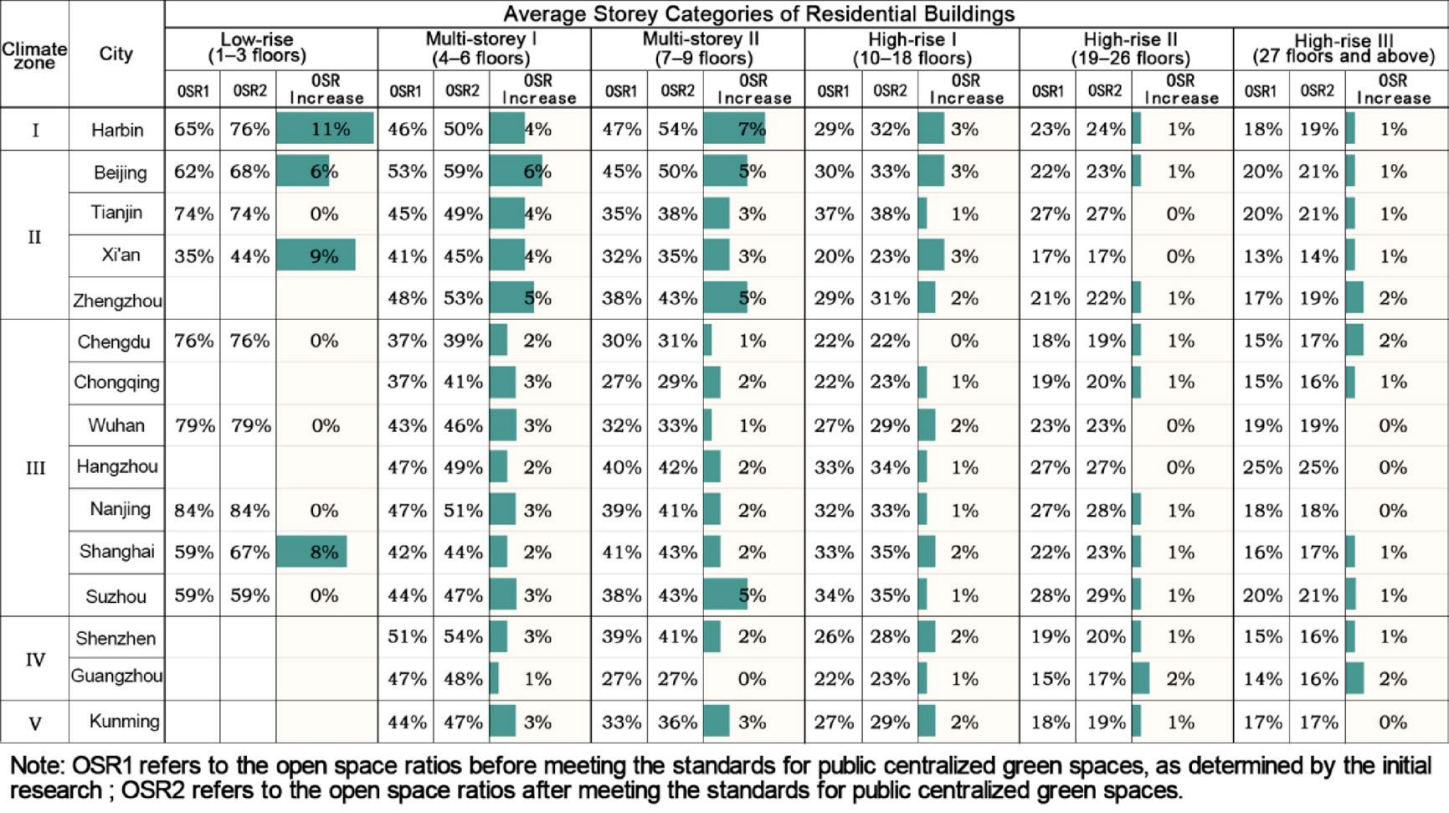
Changes in the average open space ratio of residential plots with different average storey categories in cities after meeting public centralized green space standards.

Overall, the average increase in OSR for high-rise residential plots across cities is relatively low (0–3%). The average OSR for multi-storey residential plots shows a larger increase, with cities located in Climate Zones I–II exhibiting higher increases (3–7%) than those in Climate Zones III–V (0–5%). Specifically, Harbin, located in Climate Zone I, shows a generally higher increase in the average OSR, especially for Multi-storey II plots (7%). In cities in Climate Zone II, the average increase in OSR for Multi-storey I plots (4–6%) is slightly higher than that for Multi-storey II plots (3–5%). In Climate Zone III, the analysis focuses primarily on multi-storey and high-rise residential plots, which are key components of urban development in this region. With the exception of Suzhou, where residential plots see a relatively higher increase (1–5%), other cities show more modest increases, ranging from 0 to 3%. This focus is due to the availability of more consistent data for multi-storey and high-rise buildings compared to low-rise buildings (1–3 stories), which exhibit greater variability and are less representative of urban development trends in this zone. In Climate Zone IV, the average OSR increase for residential plots in Shenzhen (1–3%) is higher than in Guangzhou (0–2%). Finally, in Climate Zone V, residential plots in Kunming show a relatively low increase in the average OSR (0–3%). (Fig. 12).

#### Open space ratio of residential plots with different building layout forms in cities

**Fig. 13:**
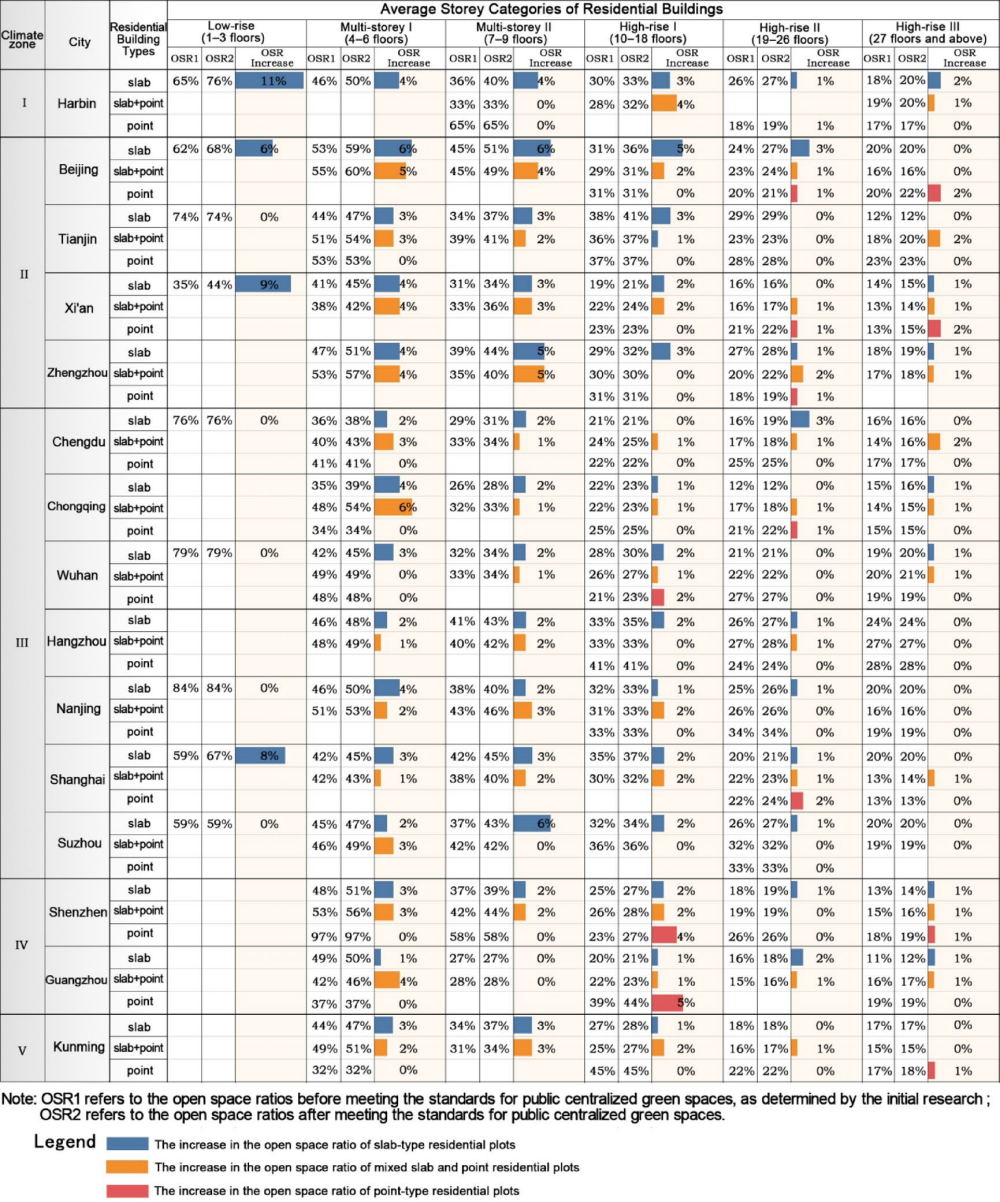
The variation in the average open space ratio of residential plots with different architectural forms across various cities after meeting the standards for public centralized green spaces.

1. Slab-type residential plots
  After meeting public centralized green space standards, slab-type residential plots in Climate Zones I–II experienced a greater increase in OSR compared to those in Climate Zones III–V. Among multi-storey slab-type residential plots: In Climate Zone I, the Multi-storey I row pattern plots and Multi-storey II perimetric pattern plots in Harbin saw a greater increase in OSR than those in cities from other climate zones. In Climate Zone II, row and hybrid pattern plots in Beijing experienced the largest increase in OSR, leading to the overall OSR increase of slab-type residential plots in Beijing being higher than in other cities in Climate Zone II. Zhengzhou followed closely behind in terms of the overall increase in OSR for slab-type plots. Although Tianjin’s overall increase was lower than other Climate Zone II cities, its perimetric pattern plots had the highest increase in OSR. In Climate Zone III, slab-type residential plots in central and western cities like Chengdu, Chongqing, and Wuhan saw a greater increase in OSR than those in cities of the Yangtze River Delta. In Climate Zone V, row pattern plots in Kunming saw a higher increase in OSR than plots with other layout methods. Among high-rise slab-type residential plots: The overall increase in OSR in Climate Zones I–II was relatively high. In particular, row and hybrid pattern plots in Beijing experienced the largest increase, while the highest increase in perimetric pattern plots was seen in Harbin. In Climate Zone III, high-rise slab-type residential plots had a generally lower increase in OSR. The highest increase for row and perimetric pattern plots was in Chengdu, while hybrid pattern plots in Nanjing and Wuhan saw relatively higher increases. In Climate Zone IV, Shenzhen’s row and hybrid pattern plots saw slightly higher increases in OSR compared to Guangzhou, while their perimetric pattern plots were similar in terms of increase (Figs. 13 and 14).

**Fig. 14:**
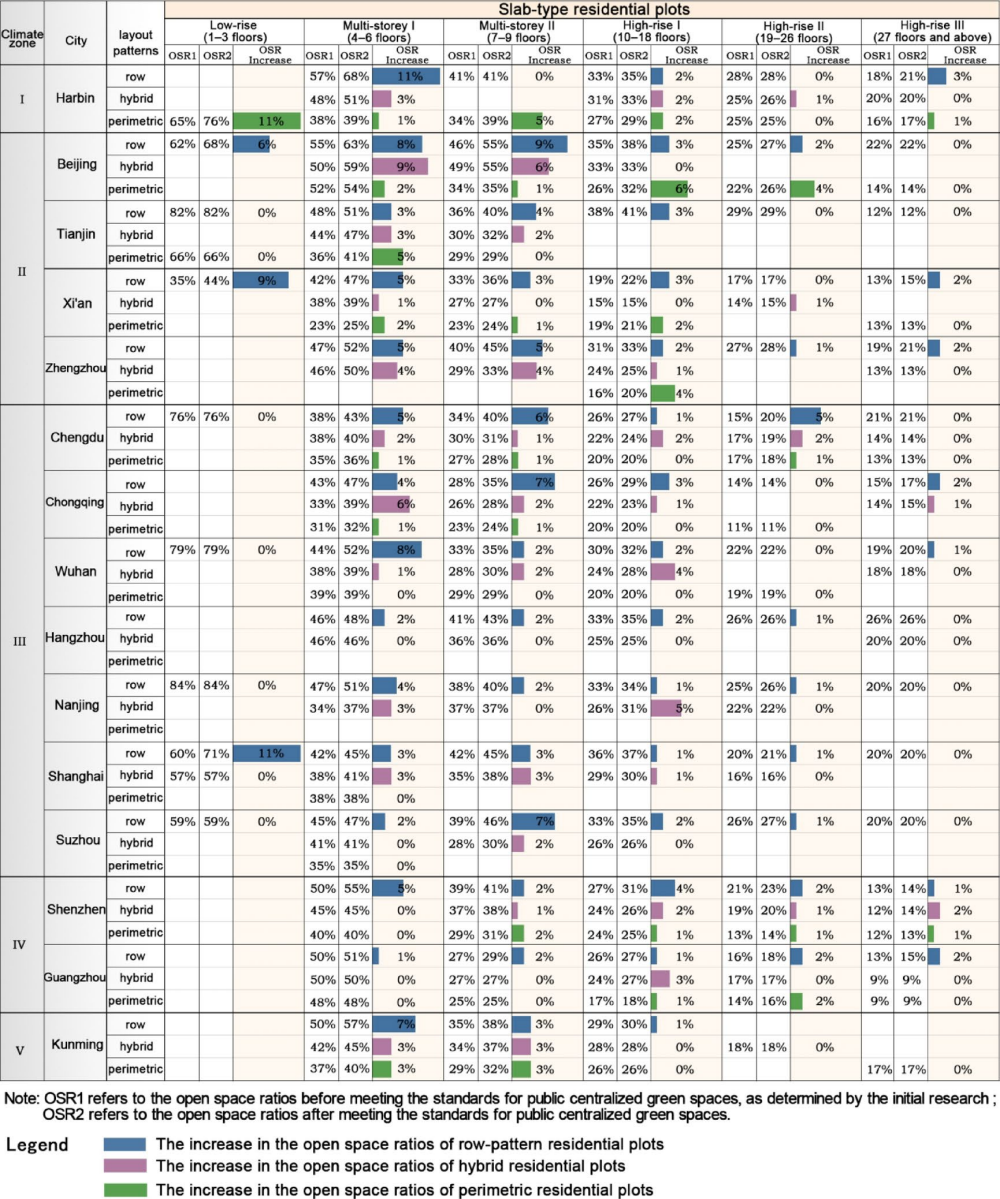
The variation in the average open space ratio of residential plots with different slab-type layout strategies across various cities after meeting the standards for public centralized green spaces.
2. Combined point-and-slab residential plots
  Among multi-storey combined point-and-slab residential plots: Cities in Climate Zone II saw a higher overall increase in OSR compared to cities in other climate zones. In particular, residential plots in Beijing with "point + row pattern" and "point + hybrid pattern" layouts experienced the largest increase in OSR, while Xi’an saw the lowest. In the central and western cities of Climate Zone III, Chongqing had the highest increase in OSR for "point + row pattern" layouts, while Chengdu saw the largest increases in "point + hybrid pattern" and "point + perimeter pattern" layouts. In cities of the Yangtze River Delta, Nanjing saw relatively higher increases in "point + row pattern" and "point + hybrid pattern" layouts, with other cities showing similar increases. In Climate Zone IV, the increase in OSR for combined point-and-slab plots in Shenzhen was higher than in Guangzhou. In Climate Zone V, the "point + perimeter pattern" layout in Kunming saw a relatively higher increase in OSR.
  Among high-rise combined point-and-slab residential plots: In Climate Zone I, the high-rise I category in Harbin with a "point + perimeter pattern" layout experienced a larger increase in OSR compared to cities in other climate zones. In Climate Zone II, cities saw similar increases in OSR for combined point-and-slab plots, but Beijing’s "point + hybrid pattern" and "point + perimeter pattern" layouts saw relatively higher increases. In Climate Zone III, the overall increase in OSR for combined point-and-slab plots was smaller. In central and western cities, Chengdu saw a greater increase in the "point + row pattern" layout, while Chongqing experienced a greater increase in the "point + perimeter pattern" layout, and Wuhan saw the largest increase in the "point + hybrid pattern" layout. Among the cities of the Yangtze River Delta, Shanghai saw a relatively higher increase in OSR. In Climate Zone IV, the increases in OSR for residential plots in Shenzhen and Guangzhou were similar (Figs. 13 and 15).

**Fig. 15:**
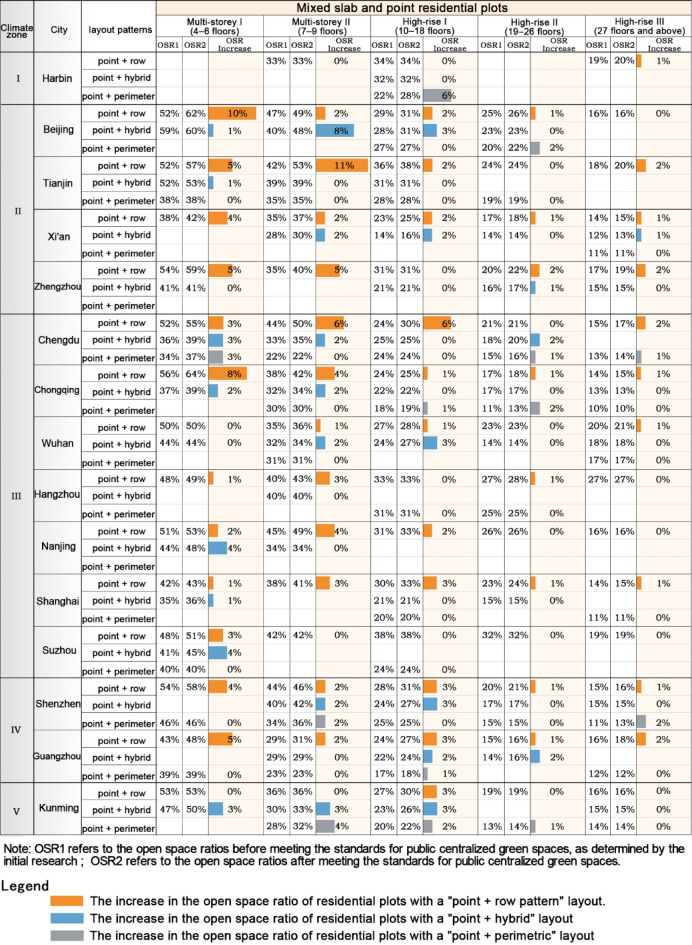
The variation in the average open space ratio of residential plots with different mixed slab and point layout strategies across various cities after meeting the standards for public centralized green spaces.
3. Point-type residential plots
  The changes in OSR for point-type residential plots in specific cities after meeting public centralized green space standards are generally small, with increases greater than 0% concentrated in high-rise categories. In the high-rise I category, Shenzhen and Guangzhou saw a higher increase in OSR for point-type residential plots compared to cities in other climate zones. In the high-rise II–III categories, Beijing, Xi’an, and Shanghai saw relatively higher increases in OSR, while other cities had almost no increase, with the rate close to 0% (Fig. 13).

#### Overall open space ratio of residential plots in cities

**Fig. 16:**
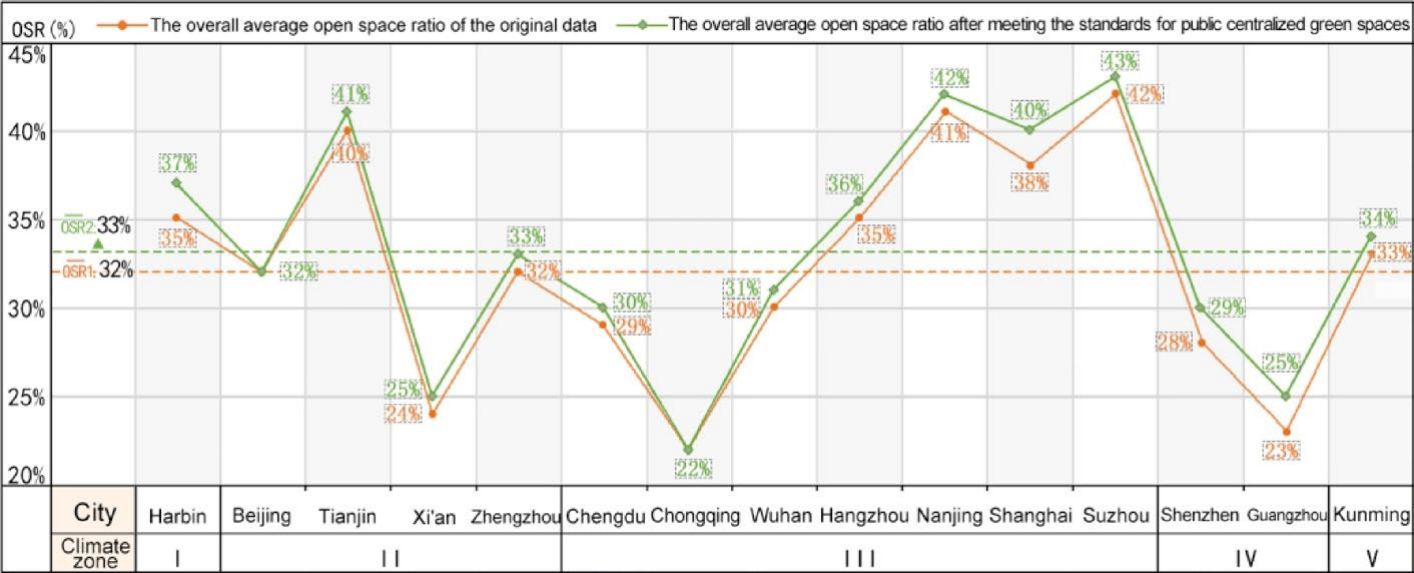
The variation in the overall average open space ratio of residential plots in various cities after meeting the standards for public centralized green spaces.

After meeting public centralized green space standards, the overall average OSR of residential plots in 15 major cities in China increased to 33%, with increases ranging between 0 and 2% across different cities. Specifically, the OSR of residential plots in Harbin, located in Climate Zone I, increased by 2%, with its overall average OSR being higher than the national average. In cities located in Climate Zone II, Beijing had the lowest increase in OSR, and the average OSRs of both Beijing and Xi’an were below the national average. In Climate Zone III, residential plots in central and western cities showed relatively lower increases in OSR, while cities in the Yangtze River Delta saw higher increases. After meeting the green space standards, the overall trend remained, with central and western cities having average OSRs below the national average, while cities in the Yangtze River Delta had average OSRs above the national average. In Kunming, located in Climate Zone V, the overall average OSR further increased after meeting the green space standards (Fig. 16).

## Discussion

### The role of open space ratio in future residential development control

The open space ratio (OSR) plays a critical role in balancing the fairness and efficiency of land development, particularly in the context of promoting sustainable urban living environments. As urbanization accelerates in China, it is essential to introduce OSR as a key indicator in land development control for residential areas^2,4,5,7^. The inclusion of OSRs helps balance land use efficiency and public interest, fosters environmental quality, addresses social and recreational needs, and aligns with policy and regulatory standards.

First, it helps balance land use efficiency and public interest: While developers prioritize maximizing land use efficiency, incorporating open space requirements into residential planning helps strike a balance between economic development and the public’s demand for accessible green spaces. This balance is crucial for ensuring the long-term sustainability and resilience of urban areas^4,5,7^.

Second, it contributes to environmental quality improvement: Open spaces are beneficial for improving air quality, alleviating urban heat island effects, and providing spaces for social activities. By integrating OSRs into planning, residential designs can foster healthier and more sustainable living environments for residents^4,18,21,48,49^.

Third, it meets social and recreational needs: As urban populations grow, the demand for public spaces, parks, and recreational areas also increases. Including OSRs ensures that residential developments prioritize providing sufficient areas for social interaction, relaxation, and outdoor activities, which are essential for community well-being^28,29,34^.

Finally, it aligns with policy and regulatory standards: Many cities around the world, including China, are increasingly implementing regulations to ensure the inclusion of public green spaces in new residential developments. As such, OSRs are becoming an important tool for guiding urban planning policies that align with environmental sustainability goals and public health standards^2,5,8^.

### Factors influencing the increase in open space ratio after meeting public centralized green space standards

Based on the characteristics of the OSR and its effective role in ensuring the quality of the living environment during residential land development, this study aims to explore an OSR indicator suitable for China’s national context. Through field surveys, interviews with urban planning authorities, and the use of national travel mapping platforms, detailed and reliable data on the basic OSRs of residential plots were obtained. Building on this data, the study further measures the OSRs after meeting the public centralized green space standards issued in China. The findings indicate that, after meeting the public centralized green space standards, the OSRs of residential plots in various cities have increased to varying degrees, suggesting that the environmental quality aspect has not yet been adequately addressed in the development and construction of residential plots in Chinese cities. The extent of the increase in the OSR of specific residential plots after meeting the public centralized green space standards is mainly influenced by the following factors:

1. Geographical location and climatic conditions: Latitude is one of the key factors determining climate zones, as it directly influences the amount of solar radiation received by a region, thereby affecting climate conditions such as temperature, rainfall, and seasonal variations. These climatic factors, in turn, have a significant impact on urban development and planning, including the layout and density of residential areas, which subsequently affects the OSR of residential plots. Compared to cities in low-latitude regions, high-latitude cities receive less solar radiation. To meet the sunlight requirements for residents, these cities typically require larger sunlight spacing between buildings, leading to wider building shadow ranges. As a result, there is less green space outside the "standard building shadow range" in public centralized green areas. Consequently, to meet the per capita green space requirements, high-latitude cities experience greater reductions in building density and more significant increases in OSRs. For instance, after meeting the green space standards, the increase in OSRs in residential plots across climate Zones I–II is generally higher than in cities located in climate Zones III–V.
2. The degree of tension in per capita construction land: In cities with limited per capita land for development, greater increases in the OSR of residential plots are needed to meet public centralized green space standards. Cities such as Beijing, Shanghai, Guangzhou, Shenzhen, and Chongqing have seen relatively higher increases in the average OSR of residential plots. Notably, both Beijing and the “Mountain City” Chongqing focus primarily on developing high-rise residential plots with higher compliance in public centralized green spaces to improve land-use efficiency^50,51^, which results in smaller increases in the overall average OSR. However, to meet public centralized green space standards, both cities have experienced relatively high increases in the OSR across different categories of residential plots. Moreover, even neighboring cities can exhibit different increases in the OSR of residential plots due to differences in land-use intensification^52^**.** For instance, although Shenzhen and Guangzhou are neighboring economically developed cities within the same climate zone, Guangzhou has a higher per capita housing area. To accommodate more residents, Guangzhou applies higher development intensity to individual residential buildings, with relatively larger building spacing, allowing for more centralized green spaces. As a result, in most categories of residential plots, the increase in OSR in Guangzhou is lower than that in Shenzhen.
3. The social and economic development status of cities: Compared to cities and regions with poorer development conditions, those with better development conditions tend to have higher environmental quality requirements, which leads to smaller increases in the OSR within the same category of residential plots. For example, in Climate Zone III, the Yangtze River Delta region, with its advantageous geographical location and better development conditions^53^, shows a greater increase in the OSR for row pattern residential plots after meeting public centralized green space standards, compared to cities in the central and western regions of the same climate zone.
4. Local construction practices: For individual cities, the increase in the OSR of residential plots after meeting public centralized green space standards is also influenced by their own construction practices. For example, in Harbin, which has a cold climate, perimetric and "point + perimeter" pattern layouts are commonly used in slab-type and combined point-and-slab residential plots^54,55^. However, these layouts in Harbin are primarily designed for "cold protection," with little consideration given to the arrangement of public centralized green spaces. As a result, the increase in OSR for perimetric and "point + perimeter" residential plots in Harbin is relatively high in order to meet green space standards. Similarly, in the Yangtze River Delta cities of Suzhou and Hangzhou, which have more favorable climate conditions, row pattern layouts are commonly used in slab-type residential plots to ensure residential comfort. However, compared to Hangzhou, Suzhou’s multi-storey row pattern residential plots have higher building densities, with most plots only meeting basic sunlight spacing requirements. While the layouts ensure good north–south ventilation, there is insufficient space for centralized green areas. Therefore, after meeting public centralized green space standards, Suzhou’s multi-storey slab-type row pattern residential plots experience a greater increase in OSR.

The sustainable urban development theory advocates for the integration of environmental, social, and economic goals in urban planning. Overall, in order to maintain the environmental quality baseline for the OSR in cities with "Chinese characteristics," it is necessary not only to meet basic sunlight spacing requirements but also to consider both “common” and “individual” factors that influence changes in the OSR of residential plots after meeting public centralized green space standards. The “common factors” refer to the varying conditions for arranging centralized green spaces in different categories of residential plots, reflecting broader principles in urban planning that emphasize the need for balanced and equitable access to open space. On the other hand, the “individual factors” include differences in building shadow range, per capita land availability, development conditions, and construction practices across different cities. These factors highlight the importance of local context in urban planning, a concept central to contextual urbanism, which emphasizes the tailoring of planning strategies to fit the specific characteristics of each urban environment. The interplay between these common and individual factors is consistent with the compact city model, which proposes that higher density development should not come at the expense of environmental quality or social well-being. In fact, achieving an optimal OSR in high-density areas is a key component of sustainable compact city design, ensuring that residents have access to necessary green open spaces despite increased building density.

## Research limitations

This study, while providing valuable insights into the OSRs of residential plots in major Chinese cities, has certain limitations.

First, the data used in this research was obtained from multiple sources, including field surveys, urban planning authority documents, and national travel mapping platforms. Due to the lack of a single, unified dataset, cross-validation was not possible. To address this limitation, the study adopted a multi-source data integration approach, continuously refining the data to enhance its authenticity and reliability. Despite these efforts, uncertainties remain, especially regarding regional variations and inconsistencies in urban planning data across different cities. These uncertainties were addressed by applying appropriate correction methods, ensuring that the findings remain robust. However, the lack of formal cross-validation leaves room for further improvement. Future research could benefit from incorporating additional validation steps, such as cross-validation using alternative datasets or methods. These steps would help to strengthen the confidence in the findings and improve the generalizability of the results. This is an important avenue for future research, which we have highlighted in the revised manuscript.

Second, in this study, representative cities were selected from five of China’s seven climate zones (Climate Zones I to V) based on their urban development characteristics, population density, and the availability of reliable data. Climate Zones VI and VII were not included for several reasons. First, reliable and consistent data for residential plots in these zones were difficult to obtain, partly due to unique political policies and considerations related to ethnic development in these regions. These factors have limited the accessibility of urban development data, making it challenging to incorporate these areas into the analysis. Second, the data for residential plots in Climate Zones VI and VII are relatively limited, which affects the ability to perform comprehensive and comparable analysis with data from other zones. These regions also exhibit unique geographical and climatic conditions, such as arid or alpine environments, which further contribute to the data availability challenges. Despite these limitations, the selected cities provide a diverse and representative sample of urban environments in China, allowing for robust analysis of OSRs across major urban regions. Future studies could expand the scope to include Climate Zones VI and VII to explore how unique political, geographical, and climatic factors influence urban development and OSRs in these regions.

Third, the study focuses primarily on the OSRs of multi-storey and high-rise residential plots, as data for low-rise buildings (1–3 stories) was sparse and exhibited higher variability. While this focus ensures robust results for the selected categories, it limits the applicability of the study to low-rise residential developments. Future research could explore low-rise residential areas in greater detail, possibly by targeting cities with more representative data on low-rise buildings or by developing new methodologies to estimate OSRs in low-rise contexts.

Finally, the study does not include a longitudinal analysis to evaluate changes in OSRs over time. Such an analysis could provide deeper insights into the long-term impact of urban planning policies and green space standards on residential developments. Future studies could incorporate longitudinal data to explore how OSRs evolve over time and examine the long-term outcomes of urban planning interventions.

Despite these limitations, the findings contribute to the understanding of OSRs in urban planning and provide a foundation for future research to address these constraints and explore additional dimensions.

## Conclusion

In China’s current land development control, there is a lack of an indicator that can directly and effectively link development efficiency factors with public interest factors. The Open Space Ratio (OSR) derived from New York City’s Zoning Ordinance has attributes that balance equity and efficiency. Although previous research has explored the introduction of OSR into China, it has yet to establish a connection between OSR and a key public interest factor—public centralized green space. To further explore a China-specific OSR, this study focuses on residential plots in 15 major cities across different climate zones in China. Using geographic data collection and technical processing, residential plots that meet public centralized green space standards were identified. Through statistical analysis and visualization, the study analyzed the compliance status of public centralized green space in urban residential plots, the overall characteristics of OSR for compliant plots, and the specific OSR characteristics of different cities. Finally, the study identified the factors that need to be considered when formulating a "China-specific" OSR that ensures residential environmental quality. The main findings of this study are as follows:

1. Currently, development control in residential plots in China’s major cities struggles to fully ensure residential environmental quality. In comparison to low-latitude cities, high-latitude cities experience significant seasonal variations in daylight hours, with long daylight periods in summer and very short days in winter. This variation presents challenges in ensuring that green spaces receive sufficient sunlight throughout the year. Meanwhile, to maximize land development efficiency, developers in high-latitude cities often reduce the size of public centralized green spaces, which in turn leads to lower compliance with green space standards. Among different residential building height categories, high-rise residential plots have a higher compliance rate than multi-storey plots. Among different residential building layout types, point-type residential plots have the highest compliance rate, followed by combined point-and-slab types, with slab-type residential plots having the lowest compliance rate. In terms of layout methods, row pattern layouts have the lowest compliance rate, while perimetric pattern layouts have the highest.
2. After meeting public centralized green space standards, the average OSR of different categories of residential plots in China’s major cities increased to varying degrees. High-rise residential plots experienced a greater increase in OSR compared to low-rise and multi-storey plots. Among building types, slab-type residential plots saw the highest increase in OSR, followed by combined point-and-slab types, while point-type plots saw the smallest increase. In terms of layout methods, row pattern layouts saw a greater increase in OSR than perimetric and hybrid layouts.
3. Although cities follow the general trend, there are significant differences across specific cities. The variation in OSR increase among cities is mainly influenced by factors such as geographic latitude, the scarcity of per capita land for development, regional economic development levels, and local construction practices.

The above findings of this study highlight the variation in OSRs across different cities in China after meeting public centralized green space standards. This demonstrates that residential land development has not yet fully addressed the creation of high-quality living environments. The practical implications of these findings are significant for local authorities. First, incorporating the OSR as a key metric in residential development control can help local governments balance land use efficiency with public interest, ensuring that economic growth does not come at the expense of accessible green spaces. This is particularly important for cities with higher development intensity, where public green spaces are often compromised. Second, local authorities can use the OSR as a tool to promote sustainable urban planning practices. By integrating OSR requirements into zoning regulations and development guidelines, governments can improve the environmental quality of urban areas, reduce urban heat island effects, and enhance public well-being. Third, the study provides a framework for local governments to evaluate and monitor the compliance of residential developments with green space standards. This can help ensure that future urban developments prioritize livability and sustainability, aligning with broader environmental and public health goals. By adopting the OSR as a regulatory and planning tool, local authorities can create more equitable and resilient urban environments that meet the needs of both current and future residents.

In summary, the application of an OSR with Chinese characteristics must be based on the premise of meeting the baseline of public interest. It should further integrate the unique developmental characteristics of each city, adopting a localized, stratified, and categorized approach to control and define OSRs. By taking the OSR as the core, an initial framework for a universally applicable development control system for residential plots can be established. This framework ensures the balance between safeguarding residents’ fundamental public interests and achieving economic efficiency in residential land development. In the absence of specific requirements, the OSR for individual residential plots can allow for multiple options. Residents would be able to enjoy basic environmental quality under any development conditions, while administrators could use the OSR as an effective tool for more efficient management. This approach ensures the protection of public interests, preserves flexibility for future development, enhances the efficiency of planning and management, and provides ample creative space for subsequent urban design work.

## Data Availability

The original contributions presented in the study are included in the article, further inquiries can be directed to the corresponding authors.
